# Travel Distance and Its Impact on Wait Time for Positron Emission Tomography–Computed Tomography in Patients with Cancers

**DOI:** 10.3390/ijerph22121816

**Published:** 2025-12-04

**Authors:** Dat T. Tran, Xiaoxiao Liu, Alka B. Patel, Rizwan Shahid, Maki Ueyama

**Affiliations:** 1 Institute of Health Economics, #4-122 Enterprise Square, 10230 Jasper Avenue, Edmonton, AB T5J 4P6, Canada; mueyama@ihe.ca; 2 School of Public Health, University of Alberta, Edmonton, AB T6G 1C9, Canada; 3 Health Intelligence, Evidence and Improvement, Primary Care Alberta, Calgary, AB T2W 3N2, Canada; xiaoxili@ucalgary.ca (X.L.); alka.patel@primarycarealberta.ca (A.B.P.); rizwan.shahid@primarycarealberta.ca (R.S.); 4 Department of Community Health Sciences, University of Calgary, Calgary, AB T2N 4Z6, Canada; 5 Department of Geography, University of Calgary, Calgary, AB T2N 1N4, Canada

**Keywords:** PET/CT, travel distance, travel time, wait time, cancer

## Abstract

To examine travel distance and its impact on wait time for positron emission tomography–computed tomography (PET/CT) in patients with lung and prostate cancers and lymphoma in Alberta. We used the Alberta cancer registry and diagnostic imaging database to identify patients with lung and prostate cancers and lymphoma who had a PET/CT scan during April 2017 and March 2023. The Alberta Facilities Distance/Time Look Up Table was used to calculate travel distance from the patient’s residence to the PET/CT facility. Negative binomial regression was used to assess the association between travel distance and wait time for PET/CT. The study included 9503 patients. Lung cancer accounted for 43.4% of the patients, followed by lymphoma (37.1%) and prostate (19.5%) cancer. There were more female patients with lung cancer (55.5%) than lymphoma (42.9%; *p* < 0.001). The mean (SD) age was 66.8 (13.8) years and lymphoma patients were younger (59.6 years) than lung (70.3 years; *p* < 0.001) or prostate (72.7 years; *p* < 0.001) cancer patients. Diabetes (14.2%) was the most prevalent comorbidity. The median (IQR) travel distance was 21 (12–121) km and this was shorter for urban (16 km) than rural (148 km; *p* < 0.001) patients, but the wait time was similar (median = 20 vs. 21 days; *p* = 0.378). There were no significant associations between travel distance and wait time (IRR = 1.00; *p* = 0.108). The results were robust in subgroup analyses by type of cancer and scan priority. There were no associations between travel distance and wait time for PET/CT. Additional research is warranted to examine the potential impact of longer travel distances on overall access to care and patient outcomes.

## 1. Introduction

Cancer is a common condition in developed countries with an aging population. The Canadian Cancer Society estimated that approximately 50% of Canadians could develop cancer during their lifetime [1]. Even though the mortality rate has decreased remarkably over time, cancer is still the leading cause of death for Canadians, and one-fourth of all deaths in Canada could be attributed to cancer [1]. The sex- and age-standardized incidence rate for all cancers was projected to decrease among males (from 464.8 to 443.2 per 100,000 population between 2003–2007 and 2028–2032) but to increase among females (from 358.3 to 371.0 per 100,000 population between 2003–2007 and 2028–2032) [2]. Due to a combination effect of continued population growth and population aging in Canada in the coming years, it was estimated that the absolute number of new cancer cases could escalate in both sexes to 280,000 cases in 2028–2032 [2,3]. Accordingly, the economic burden of cancer care in Canada increased from CAD 3.7 billion (in 2024 CAD) in 2005 to CAD 8.3 billion (in 2024 CAD, equaling 3.6% of total health expenditure) in 2012, and is expected to increase further in the future [4,5,6].

Alberta has an integrated, publicly funded, and universally covered healthcare system serving a population of over four million people in five health zones in a large and diverse geographical area (Supplementary Figure S1). The cancer epidemic in the province has been projected to follow suit with the national trends. One in two people may develop a cancer during their lifetime, of which breast (for women), prostate (for men), lung, and colorectal cancers are the most prevalent [7]. Lymphoma is also quite common in both men and women [7,8]. The total number of cancer cases in the province has been projected to reach 22,000 in 2021 (approximately 482 cases per 100,000 population), representing a 115% increase from 1996 [7]. The total healthcare costs related to cancer in Alberta were previously estimated at CAD 623.6 million (in 2024 CAD) per year [5,9].

Positron emission tomography–computed tomography (PET/CT) is considered an emerging clinical diagnostic imaging technique that could greatly assist in the diagnosis and treatment of several diseases, including cancer. PET/CT is currently recommended for initial staging, restaging, and treatment response assessments in patients with lung and prostate cancers and lymphoma in Canadian, American, and European guidelines [8,10]. Presently, there are four PET/CT facilities with five scanners located in the two biggest urban centers in Alberta. There is one scanner each in the Cross Cancer Institute, Royal Alexandra Hospital, and the University of Alberta Hospital in the city of Edmonton, while the Foothills Medical Centre in the city of Calgary has two scanners (Supplementary Table S1 and Figure S1). Patients with a PET/CT order may need to enter a booking system and join a waitlist for their scan. However, there is no single central booking system for PET/CT in Alberta, so the facility of choice is dependent on physician and patient preference, travel distance, and availability and wait time at the target scanning facility. Generally, the Cross Cancer Institute can receive patients from anywhere in the province. Patients from the South and Calgary zones mostly have PET/CT scans at the Foothills Hospital, while patients in the North and Edmonton zones can have services at the University of Alberta Hospital or Royal Alexandra Hospital. Patients in the Central zone can go to either facility based on its availability and corresponding wait time. AHS recommends a target wait time for PET/CT ranging from 2 to 6 weeks depending on the priority of the scan, which includes priorities 1 (urgent), 2 (semi-urgent), 3 (not urgent), and 4 (scheduled exams). There are no wait time targets for scheduled exams as they are determined by the physicians according to their clinical decision and the patients do not have to enter the waitlist for a PET/CT scan (Supplementary Table S2) [11].

Previous studies have suggested that increased travel burden may contribute to delayed diagnosis and treatment, reduced treatment adherence and health outcomes, increased disparity in healthcare access, and increased financial strains on patients with cancer [12,13,14]. For example, Rocque et al. (2019) reported higher spending and hospitalization rates for Medicare patients with cancer who had to travel longer than 1 h in the southeastern United States [13]. In a scoping review of the travel burden and financial hardship in cancer care in 2024, Planey et al. found that those who encountered transportation challenges were more likely to forgo or discontinue needed care [12]. A recent systematic review (2024) on the relationship between travel distance and patient outcomes in radiotherapy by Silverwood et al. found mixed results, where 5 out of the 16 included studies reported negative impacts, 4 studies reported positive impacts, and 7 studies found no significant association between a longer travel distance and patient survival [14]. There is a gap in the current literature on travel distance from patient’s residence to PET/CT facility and wait time for the scanning procedure for patients with cancers in Alberta. Accordingly, we evaluated travel distance and wait time for PET/CT, and whether travel distance could have an impact on wait time for the procedure in patients with lung and prostate cancers and lymphoma. Lung and prostate cancers and lymphoma were selected in response to the provincial priorities. The study findings could support policy decision-making on the capacity, demand, supply, and use of PET/CT in patients with cancers in Alberta.

## 2. Methods

### 2.1. Data Source and Study Population

We conducted a population-based retrospective cohort study using the Alberta Cancer Registry (ACR) and Alberta administrative datasets to include patients aged ≥18 years with lung and prostate cancers and lymphoma who were in the ACR and used PET/CT between 1 April 2017 and 31 March 2023 (the study period). The ACR contains a complete register of patients diagnosed or treated with cancer in Alberta from 1982 and provides rich data on patients, such as demographics, referrals to medical oncologists, tumor topography and morphology at diagnosis, and treatments during patient follow-up [15]. The North American Association of Comprehensive Cancer Registries has recognized the excellent quality of the ACR data [16].

The ACR was linked to other Alberta administrative health datasets, including the diagnostic imaging (DI) database and population registry using unique personal healthcare numbers [17]. The DI database records all DI encounters for patients (e.g., X-Ray or PET/CT) and the population registry provides demographics for all inhabitants of Alberta who are members of the Alberta Health Care Insurance Plan [17,18,19].

### 2.2. Variables of Interest

Travel distance and time from a patient’s residence location to a PET/CT facility was based on the “Alberta Facilities Distance/Time Look Up Table” by Alberta Health Services (AHS) Applied Research and Evaluation Services [20]. Briefly, the distance along a road network (by car) traveled from a patient location (by postal code) to a healthcare facility (by exact location) were calculated using multiple linked datasets, including the Postal Code Translation File, DMTI Route Logistics Road Network File, Alberta Municipality Data Sharing Partnership road data, and AHS Facility Locations. The calculation considered several factors potentially affecting travel distance and time, such as one-way road, primary/secondary road (with posted speed limit), and winter travel. The road network model was validated using actual travel times from emergency medical service interfacility transfer (non-urgent) data. An optimal scenario (with posted speed limits and no delays due to rush hour, road closure, or traffic accidents) was used to generate travel distance and time. The method of calculating travel distance and travel time has been used previously [21].

The wait time for a PET/CT scan was defined as the time from the order (or booking) date, when the physician and patient agreed to the PET/CT scan, to the service date, when the PET/CT scan was performed. Because the PET/CT procedures could also be used for non-cancer conditions, we only considered the PET/CT scans ordered after the date of cancer diagnosis (incidence), assuming that these PET/CT scans were for cancers. We followed wait time computation methods from Canadian Institute for Health Information and AHS in which we excluded scans that were for inpatient/emergency patients (7.5% of the study cohort) because these patients usually did not have to wait and there is no target wait time for this patient group. We also excluded a scanning encounter where the patient deferred the planned scan (5.7% of the study cohort) [11,22].

### 2.3. Statistical Analysis

Patient characteristics and unadjusted outcomes (i.e., travel distance or wait time) were reported as mean (standard deviation), median (interquartile range), and count (proportion), as appropriate. A *t*-test and Kruskal–Wallis test were used for continuous variables, and the χ^2^ test was used for categorical variables, respectively. Patient median household income in the residential neighborhood (forward sortation area level) was based on the 2021 Canada Census (provided by Alberta Health), while residency (urban or rural) was based on the second digit of the postal code [23]. Charlson comorbidities were examined and included in the analyses because they could impact healthcare service access and use [24,25]. Previously validated International Classification of Diseases codes were used to identify patient comorbidities [26], which were considered to be present if they were recorded in the Discharge Abstract Database or National Ambulatory Care Reporting System in the one year before the PET/CT scan order date.

We calculated travel distance and wait time for both the first PET/CT scan and subsequent scans during the study period. We excluded scheduled exams from the models examining the risk-adjusted association between travel distance and wait time because the wait time of the scheduled exams was pre-determined by physicians. Further, we only included the first scan in the model because initial data examination indicated that the travel distance and wait time were found to be similar between the first scan and subsequent scans, and 71.3% of the subsequent scans were scheduled exams. Therefore, adding subsequent scans would not improve the models while it could introduce additional bias due to within-patient interactions. In addition to a model for the whole study cohort, we performed subgroup analyses where we evaluated adjusted associations between travel distance and wait time by type of cancer and for urgent and semi-urgent scan priorities, which accounted for the majority of the scans.

We used multivariable negative binomial regressions to assess the association between travel distance and wait time. The primary variables of interest were travel distance (in units of 10 km), patient sex, age, type of cancer, residence location (urban or rural), and health zone. We first included the primary variables and additional risk factors in the model and used a backward stepwise variable selection process with a Likelihood Ratio (LR) test to examine retainment of the additional risk factors in the final regression model. The additional risk factors included household income quartiles, Charlson comorbidity score, scan priority, scan year, number of tumors, cancer stage at incidence, time from the cancer incidence to the PET/CT order date, whether the patient had another cancer, and the scanning facility. Except for the primary variables, a variable remained in the final regression model if the LR test was significant at a 10% level. We did not use the traditional stopping rule of a 5% significant level because it has been reported that a strict rule could lead to the exclusion of important variables [27,28,29]. The remaining variables were checked for collinearity using a correlation matrix.

All analyses were performed using SAS Studio 3.8 (SAS Institute, Cary, NC, USA) and Stata version 14 (Stata Corporation, College Station, TX, USA); two-sided *p*-values < 0.05 were considered statistically significant.

### 2.4. Sensitivity Analysis

We examined the association between travel distance and wait time for PET/CT scans in the base case analysis. We performed another modeling exercise to assess if there could be an association between travel time and wait time.

The study period coincided with the Coronavirus 2019 (COVID-19) pandemic, in which public health restrictions were introduced on 11 March 2020 in Alberta [30]. It has been reported previously that there were significant declines in hospital admissions and ED visits during the pandemic in the province [31]. Therefore, we compared PET/CT wait time prior to the pandemic (ordered before 11 March 2020) and during COVID-19 (ordered 11 March 2020 onwards) to examine whether COVID-19 had an impact on PET/CT wait times.

We excluded scheduled exams from the modeling to examine association between travel distance and wait time in the main analysis because the wait time for scheduled exams is pre-determined by physicians. We conducted additional analyses where we included all PET/CT scans because a patient’s travel distance might be a factor to consider when a physician decides on when they need results of the scheduled exams.

## 3. Results

### 3.1. Patient Characteristics

**Figure 1:**
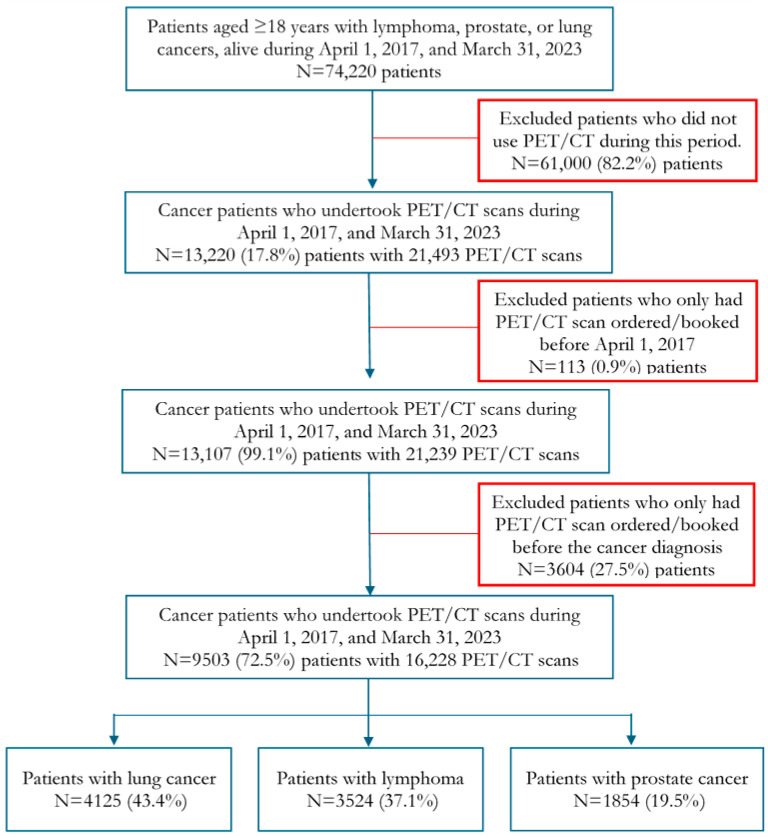
Patient selection flowchart.

There were 74,220 unique patients with lung and prostate cancers and lymphoma in Alberta who were 18 years or older at the time of cancer diagnosis and alive at least one day between 1 April 2017 and 31 March 2023 (the study period). After excluding patients who did not undertake a PET/CT scan, who had a PET/CT scan ordered before the start of the study period, and patients who had a PET/CT scan ordered before the cancer diagnosis, the final study cohort included 9503 (12.8%) patients who undertook 16,228 PET/CT scans during the study period. Of them, patients with lung cancer accounted for 43.4%, followed by lymphoma (37.1%) and prostate (19.5%) cancer. Patient selection is depicted in Figure 1.

Patient characteristics at the first PET/CT scan request are presented in Table 1. There were more female patients with lung cancer (55.5%) than with lymphoma (42.9%; *p* < 0.001). The mean (SD) age was 66.8 (13.8) years and patients with lymphoma (59.6 years) were younger than those with lung (70.3 years; *p* < 0.001) or prostate (72.7 years; *p* < 0.001) cancers. Eighty-four percent of the patients resided in urban areas.

Diabetes (14.2%) and chronic pulmonary disease (11.1%) were the two most prevalent comorbidities, and they were more common in patients with lung and prostate cancers than those with lymphoma, especially for chronic pulmonary disease where almost one in five patients with lung cancer had it (18.3%). Both diabetes (16.2% vs. 13.8%; *p* = 0.013) and chronic pulmonary disease (15.4% vs. 10.3%; *p* < 0.001) were also more prevalent in patients who resided in rural areas compared to those in urban areas. Patients with lung cancer had a higher Charlson score (mean = 3.7) than patients with lymphoma (mean = 2.9; *p* < 0.001) and patients with prostate (mean = 3.1; *p* < 0.001) cancers. However, there were no differences in Charlson score between urban (mean = 3.3) and rural (mean = 3.4; *p* = 0.165) patients.

Patients residing in the Calgary zone (35.2%) and Edmonton zone (34.8%) accounted for 70% of the patients, while the South zone (6%) contributed the least patients. Of the four PET/CT facilities in Alberta, Foothills Hospital led the service provision with 45.6%, followed by the Cross Cancer Institute with 35.6% (Table 1).

### 3.2. Travel Distance

Overall, a patient with cancer traveled a median (IQR) of 21 (12–121) km to receive a PET/CT scan. The travel distance was shorter for patients in urban areas (median = 16 km) than those in rural areas (median = 148 km; *p* < 0.001). Patients in Calgary and Edmonton zones only traveled a fraction of the distance (16 and 14 km, respectively) of their counterparts in the Central (140 km), North (282 km), and South (215 km) zones, because all four PET/CT facilities are located in the two respective cities (Table 2).

Travel time in optimal conditions (e.g., no roadblocks and at optimal speed) to PET/CT facilities is presented in Supplementary Table S3. Overall, a patient spent a median of 40 min to go for a PET/CT scan in Alberta. The travel time also greatly varied between urban (36 min) and rural (121 min; *p* < 0.001) areas and across health zones (a minimum of 31 min in Edmonton to a maximum of 203 min in the North zone; *p* < 0.001). Although the travel time also differed between facilities (*p* < 0.001), the difference was only 10 min (a minimum of 34 min at the University of Alberta Hospital to a maximum of 44 min at the Cross Cancer Institute).

### 3.3. Wait Time for PET/CT

Of the 9503 first PET/CT scans during the study period, 712 (7.5%) were from inpatient/emergency units and were excluded from wait time calculations. Of the remaining 8791 outpatient patient scans, we further excluded 505 (5.7%) who requested a delay in scanning or if an indication of priority was not available, resulting in a cohort of 8286 patients for wait time calculations. There were no differences between the wait time patient cohort and patients who delayed the scan (excluded) with regard to sex, age, residence location, median household income, and Charlson comorbidity score. However, close to half of the delayed patients were those who resided in the Edmonton zones (47.7%) (Supplementary Table S4).

The median (IQR) wait time was 20 (11–30) days. The wait times for patients with lung cancer (median = 19 days) and lymphoma (median = 20 days) were shorter than those for patients with prostate cancer (median = 25 days; *p* < 0.001) (Table 3). Although the travel distance greatly differed between urban and rural areas, the wait time was similar between the two regions (20 days vs. 21 days; *p* = 0.378). Similarly, there were no differences in wait time between the five health zones in Alberta (*p* = 0.162) despite a substantial disparity in travel distances for patients residing in these health zones (Table 2). Although there were variations in wait times between facilities, the wait times met AHS target recommendations for patients with urgent and semi-urgent priorities who accounted for 96.4% of the patients who had to enter the waitlist, and where wait time is subjected to an AHS target (Table 4).

Before risk adjustment, there were no significant associations between travel distance and wait time for the first PET/CT scan in Alberta (IRR = 1.00; *p* = 0.852). After adjusting for patient and regional characteristics, there were no significant associations between travel distance and wait time (IRR = 1.00; *p* = 0.108) for the first scan (Table 5). The adjusted wait time was similar for rural and urban areas (IRR = 1.00; *p* = 0.943). Compared to patients who resided in the Calgary zone, the wait time for PET/CT was similar for patients living in the Central (IRR = 1.00; *p* = 0.952), Edmonton (IRR = 0.99; *p* = 0.819), North (IRR = 1.01; *p* = 0.891), and South (IRR = 1.02; *p* = 0.607) zones. Patients at the Foothills (IRR = 1.25; *p* < 0.001), Royal Alexandra (IRR = 1.37; *p* < 0.001), and University of Alberta (IRR = 1.88; *p* < 0.001) hospitals had a longer wait time than those who attended the Cross Cancer Institute (Supplementary Table S5).

Subgroup analyses by scan priority and type of cancer showed similar results. There were no associations between travel distance and wait time in patients with urgent (IRR = 1.00; *p* = 0.263) and semi-urgent (IRR = 1.00; *p* = 0.109) scan priorities, and in patients with lung cancer (IRR = 1.00; *p* = 0.429), lymphoma (IRR = 1.00; *p* = 0.935), and prostate cancer (IRR = 1.00; *p* = 0.236) (Table 5). Detailed results of negative binomial regressions assessing associations between travel distance and wait time for PET/CT by scan priority and by type of cancer are presented in Supplementary Tables S6–S7 and S8–S10, respectively.

### 3.4. Sensitivity Analysis

Similarly to travel distance, travel time had no significant association with wait time for PET/CT scans for all patients (IRR = 1.00; *p* = 0.159). The results were robust in two analyses by scan priorities (urgent and semi-urgent) and three analyses by type of cancer (lung, lymphoma, and prostate) (Supplementary Table S11).

There were no differences in PET/CT wait time during the pre-pandemic (20 days) and COVID-19 (21 day; *p* = 0.209) periods. The wait time was also similar between the pre-pandemic and COVID-19 periods in urban (20 vs. 21 days; *p* = 0.403) and rural (21 vs. 20 days; *p* = 0.200) areas (Supplementary Table S12).

The results of sensitivity analyses with the addition of scheduled exams (priority 4) are presented in Supplementary Table S13. The trends remained without association between travel distance and wait time for all patients (IRR = 1.00; *p* = 0.107), and patients with lung cancer (IRR = 1.00; *p* = 0.950) and lymphoma (IRR-1.00; *p* = 0.823). However, a 10 km longer travel distance was associated with a 1% increase (or 0.2 days) in wait time (IRR = 1.01; *p* = 0.034) in patients with prostate cancer. Further, a 10 km longer travel distance was found to be significantly associated with a 1% decrease (approximately 0.7 days) in wait time (IRR = 0.99; *p* = 0.019) in patients with a non-urgent (priority 3) priority, though the sample size was small (234 patients).

## 4. Discussion

Our population-based retrospective cohort study of patients with lung and prostate cancers and lymphoma in Alberta, who ordered and had a PET/CT scan between April 2017 and March 2023, found that a majority of the patients resided in the Calgary and Edmonton zones, and that they received PET/CT scans mainly from the Foothills Hospital (45.6%) and Cross Cancer Institute (35.6%). The other two PET/CT facilities (the University of Alberta and Royal Alexandra hospitals) provided a smaller share of the PET/CT services in the province for patients with these three cancers. There was substantial variation in travel distance for PET/CT scans between patients in urban areas (16 km) and rural areas (148 km), as well as across health zones (ranging from 14 to 282 km). However, the wait time was similar for patients in urban areas (20 days) and rural areas (21 days). Wait time was almost the same across health zones (20–21 days). The wait time for PET/CT met AHS target wait time recommendations for urgent and semi-urgent priority patient groups, who accounted for 96.4% of the patients who had to enter the waitlist for PET/CT. After risk adjustments, we did not find any associations between travel distance and wait time. The results were robust in all subgroup and sensitivity analyses including using travel time as a proxy for travel burden. There was one exception, a positive association was observed between travel distance and wait time in patients with prostate cancers when scheduled exams (not subject to entering a waitlist) were taken into consideration.

The findings on no significant association between travel distance and wait time have important implications. First, this suggests that Alberta DI services for the three cancers considered in this study were delivered effectively with regard to wait time during the study period. The contrast between a similarity in wait time and a vast difference in travel distance may also suggest efficient and effective coordination between physicians, DI services, and patients. Further, the findings suggest that travel distance was generally not a barrier to meeting PET/CT wait time guidelines and access to care for an average patient with lung cancer, lymphoma, or prostate cancer in Alberta. Therefore, additional PET/CT capacity (e.g., mobile PET/CT to rural and remote regions) may potentially reduce travel distances needed for some patients, but it may not have an impact on the overall wait time for PET/CT in Alberta. Nonetheless, long waits for diagnostic procedures could be a source of distress in patients with cancer; therefore, reducing travel distance could still support the patient’s fight against cancer and improve patient satisfaction, which is an important factor in a patient-centered healthcare system [32].

Travel distance could serve as a behavioral measure of patient preference and our findings of insignificant impact of travel distance on wait time further emphasized that travel distance (and travel time) may have complex effects on access to care and patient outcomes depending on the healthcare systems, localities, populations, and healthcare specialties [33,34]. In non-cancer-specialized care in the United States, Visingardi et al. (2025) found no impact of increased travel distance on the risk of intervention in patients with renal trauma [35], while Telfeian et al. (2025) suggested that patients were willing to travel long distances to receive specialty endoscopic spine surgery [36]. In contrast, Gupta et al. (2025) examined primary care use in Ontario, Canada, and reported that patients who lived >30 km from their family physician were less likely to have family physician visits and screening for colon, breast, and cervical cancers [37]. Likewise, Liu et al. (2022) evaluated travel distance and time for patients with osteoarthritis in Alberta, Canada, to general practitioners, orthopedic surgeons, and physiotherapists, and reported a significant disparity in realized access to healthcare providers between urban and rural areas [21]. These studies could suggest the impact of travel distance in specialized care might not be as significant as that in non-urgent primary care settings, although the differences in healthcare systems could play a role in how patients access needed health services, as reported previously [38].

In cancer care, a longer travel distance may have a more consistent impact on healthcare access in poorer localities. For example, a systematic review by Elattabi et al. (2025) found that a longer travel distance was significantly associated with advanced cancer stages at diagnosis in six sub-Saharan African countries (Ethiopia, Namibia, Nigeria, Uganda, Zambia, and Botswana) but had no impacts in Scotland, Canada, and the United States [39]. Another study at a Rwandan cancer center that serves a low-income rural population also found higher odds of late-stage diagnosis among patients living in the farthest distance quartile [40]. In radiotherapy for patients with cancer, a systematic review by Silverwood et al. (2024) reported positive, negative, and no impacts of travel distance on adherence, receiving guideline-concordance therapy, and survival in Australia, Canada, Greece, and the United States [14]. Also, Myneni et al. (2025) reported the low use of ancillary services (e.g., diet or pain management consultation) in patients with pancreatic cancers with long travel distances in a single center in Maryland, United States [41].

Although we found consistent wait times for PET/CT regardless of significant variations in travel distance in this study, it should be noted that wait times for PET/CT are only part of the overall wait times for treatment and care, and there could be multiple factors throughout the care pathways that could impact the overall time to treatment. For example, Stokstad et al. suggested some common reasons for treatment delays, namely duplication in procedures, newly discovered issues that need additional sequential diagnostic procedures, pathology reports not acted upon in a timely manner, late referrals, and long wait times for diagnostic procedures [42]. Thus, further reducing PET/CT wait times (e.g., by providing mobile PET/CT services), which largely met target recommendations for this specific patient population, may not have an impact on patient outcomes as well as the healthcare system in Alberta. However, adding additional PET/CT capacity could still potentially have benefits for other diseases that need PET/CT, such as cardiovascular disease, neurological conditions, or other cancers. Nonetheless, an effort to identify and address other potential roadblocks in access to treatment and care may provide benefits to patients with cancer in Alberta.

Timely access to necessary care is an important metric for quality of care. The literature of the impact of wait time on patient outcomes in cancer care is not rich, not fully conclusive, and is changing over time. To our knowledge, most studies focus only on lung cancer. Several studies dated in the 2000s reported negative or no associations between wait time and patient outcomes in patients with lung cancer [43,44,45,46,47,48], while more recent studies suggested that long wait times in general could be considered to have a negative impact on patient outcomes in addition to a being a source of increased distress in patients [32]. For example, a study by Mohamed et al. in 2011 reported a positive association between treatment delay and disease progression in patients with lung cancer in the United States, which was also found in another study by Everitt et al. in 2013 in Australia [49,50]. A more recent multi-center international prospective cohort study with a larger sample size in 2021 that used the time interval between staging and radiotherapy planning PET/CT scans (median = 42 days) as an exposure metric, concluded that long intervals between PET/CT imaging and treatment initiation were associated with a higher rate of progression in patients with non-small-cell lung cancer [51]. The differences in findings could stem from differences in the healthcare systems and advancements in health technology in recent decades, where cancer could now be diagnosed earlier and new treatments could have more powerful impacts on patient survival if provided in a timely manner [52,53].

Although this study provides novel data on travel distance and wait time for PET/CT, and the association between travel distance and wait time, it has several limitations. First, this study only included patients who had a PET/CT scan, so patients who were ordered a PET/CT scan but did not survive long enough to receive the scan, were not included in the assessment. However, given the short wait time for PET/CT, the impact of deceased patient exclusion, if any, should be minimal. Further, it should be noted that PET/CT can also be used for other conditions (e.g., other cancers, cardiovascular, and neurological diseases) and we could not account for the impact of the waitlist of PET/CT for these conditions on the wait time for PET/CT in patients with cancers in the present study because of data unavailability. Similarly, we did not have data on other patient risk factors such as race or Indigenous status. In addition, we used travel distance and travel time along a road network (by car) in Alberta where 86.6% of households have a car and it is used as the main travel means by 87.3% of the employed workforce in Canada [54,55]. Cautions should be taken into consideration when translating results into jurisdictions where other types of travel like rail or ferry have a bigger role in the everyday commute.

## 5. Conclusions

Our population-based retrospective cohort study of patients with lung cancer, lymphoma, and prostate cancer in Alberta found that there were generally no associations between travel distance and wait time for PET/CT in this population despite a large disparity in travel distances and travel times across geographic regions. Our study findings highlight the potential complex impact of geographic location on access to care and could be used for healthcare service planning. Additional research is warranted to examine if additional PET/CT capacity (e.g., mobile PET/CT) could have an impact on other groups of patients (e.g., patients with cardiovascular disease or other cancers) in Alberta.

## Figures and Tables

**Table 1:** Patient characteristics at the first PET/CT scan request.

Variable	All Patients	Lung	Lymphoma	Prostate	*p*
Patients, *n* (%)	9503	4125 (43.4)	3524 (37.1)	1854 (19.5)	
Number of PET/CT scans, *n* (%)	16,228	5497 (33.9)	8026 (49.5)	2705 (16.7)	
Females, *n* (%)	3801 (40)	2289 (55.5)	1512 (42.9)	—	<0.001 *
Age, in years, mean (SD)	66.8 (13.8)	70.3 (9.9)	59.6 (16.6)	72.7 (8.9)	<0.001
Age, in years, median (IQR)	69 (60–76)	71 (64–77)	62 (50–72)	73 (66–79)	<0.001
Age group, *n* (%)					
18–49 years	980 (10.3)	104 (2.5)	864 (24.5)	12 (0.7)	<0.001
50–59 years	1184 (12.5)	440 (10.7)	617 (17.5)	127 (6.9)	
60–69 years	2727 (28.7)	1280 (31)	923 (26.2)	524 (28.3)	
70–79 years	3163 (33.3)	1589 (38.5)	820 (23.3)	754 (40.7)	
≥80 years	1449 (15.3)	712 (17.3)	300 (8.5)	437 (23.6)	
Urban residence, *n* (%)	7981 (84)	3432 (83.2)	3023 (85.8)	1526 (82.3)	<0.001
Household income in CAD, mean (SD)	96,199 (34,972)	90,920 (31,791)	100,215 (36,536)	100,291 (37,071)	<0.001
Patient type at scanning, *n* (%)					
Outpatient	8791 (92.5)	3875 (93.9)	3160 (89.7)	1756 (94.7)	<0.001
Inpatient/Emergency	712 (7.5)	250 (6.1)	364 (10.3)	98 (4.3)	
Health zone, *n* (%)					
Calgary	3340 (35.2)	1435 (34.8)	1323 (37.5)	582 (31.4)	<0.001
Central	1330 (14)	643 (15.6)	439 (12.5)	248 (13.4)	
Edmonton	3308 (34.8)	1371 (33.2)	1203 (34.1)	734 (39.6)	
North	956 (10.1)	417 (10.1)	330 (9.4)	209 (11.3)	
South	569 (6)	259 (6.3)	229 (6.5)	81 (4.4)	
PET/CT facility, *n* (%)					
Cross Cancer	3379 (35.6)	1466 (35.5)	1501 (42.6)	412 (22.2)	<0.001
Foothills	4336 (45.6)	1928 (46.7)	1704 (48.4)	704 (38.0)	
Royal Alex *	593 (6.2)	297 (7.2)	60 (1.7)	236 (12.7)	
U of A	1195 (12.6)	434 (10.5)	259 (7.4)	502 (27.1)	
Comorbidities, *n* (%)					
Myocardial infarction	169 (1.8)	76 (1.8)	58 (1.7)	35 (1.9)	0.749
Heart failure	347 (3.7)	177 (4.3)	100 (2.8)	70 (3.8)	0.003
Peripheral vascular disease	222 (2.3)	143 (3.5)	39 (1.1)	40 (2.2)	<0.001
Cerebrovascular disease	259 (2.7)	139 (3.4)	64 (1.8)	56 (3)	<0.001
Chronic pulmonary disease	1055 (11.1)	754 (18.3)	172 (4.9)	129 (7)	<0.001
Dementia	121 (1.3)	44 (1.1)	18 (0.5)	59 (3.2)	<0.001
Rheumatoid disease	156 (1.6)	69 (1.7)	74 (2.1)	13 (0.7)	<0.001
Liver disease	177 (1.9)	83 (2)	76 (2.2)	18 (1)	0.006
Diabetes	1348 (14.2)	612 (14.8)	459 (13)	277 (14.9)	0.045
Renal disease	297 (3.1)	109 (2.6)	110 (3.1)	78 (4.2)	0.006
Hemiplegia/Paraplegia	43 (0.5)	19 (0.5)	15 (0.4)	9 (0.5)	0.948
HIV/AIDS	20 (0.2)	4 (0.1)	15 (0.4)	1 (0.1)	0.002
Charlson comorbidity score, mean (SD)	3.3 (2.3)	3.7 (2.5)	2.9 (1.9)	3.1 (2.1)	<0.001

Notes: CAD = Canadian dollars; HIV/AIDS: human immunodeficiency virus/acquired immunodeficiency syndrome; IQR: interquartile range; *p* = *p*-value; SD = standard deviation. * Between lung and lymphoma patients only. Cross Cancer = Cross Cancer Institute; Foothills = Foothills Hospital; Royal Alex = Royal Alexandra Hospital; U of A = University of Alberta Hospital. * Royal Alexandra Hospital started providing PET/CT in 2021.

**Table 2:** Travel distance (in km) to the first PET/CT scanning (*n* = 9503 patients).

Variable, Median (IQR)	All Patients	Lung	Lymphoma	Prostate
Overall	21 (12–121)	21 (12–132)	20 (12–110)	20 (12–204)
Urban	16 (11–43)	16 (10–54)	17 (11–39)	17 (10–36)
Rural	148 (83–224)	147 (85–224)	167 (83–226)	144 (83–222)
Health zone				
Calgary	16 (11–27)	16 (11–26)	16 (11–27)	17 (11–29)
Central	140 (91–154)	141 (93–153)	141 (91–160)	133 (87–152)
Edmonton	14 (9–19)	13 (8–18)	14 (9–20)	14 (9–19)
North	282 (146–451)	258 (146–451)	288 (168–451)	242 (123–447)
South	215 (209–293)	224 (210–293)	214 (209–293)	213 (202–293)
PET/CT facility				
Cross Cancer	26 (12–134)	31 (12–143)	20 (12–129)	30 (12–130)
Foothills	21 (12–98)	21 (13–130)	21 (12–87)	19 (12–63)
Royal Alex	18 (9–131)	18 (8–132)	19 (10–129)	18 (12–129)
U of A	16 (10–100)	15 (10–68)	16 (11–86)	22 (11–123)

Notes: Cross Cancer = Cross Cancer Institute; Foothills = Foothills Hospital; IQR = inter-quartile range; Royal Alex = Royal Alexandra Hospital; U of A = University of Alberta Hospital.

**Table 3:** Wait time (in days) at the first scan for outpatient patients with cancer in Alberta (*n* = 8286).

Variable, Median (IQR)	All Patients	Lung	Lymphoma	Prostate
Overall	20 (11–30)	19 (11–27)	20 (8–34)	25 (14–49)
Residence location				
Urban	20 (11–31)	19 (11–27)	19 (8–34)	25 (14–51)
Rural	21 (12–30)	19 (11–26)	21 (11–37)	23 (15–42)
Health zone				
Calgary	21 (10–29)	19 (10–25)	21 (9–34)	25 (14–49)
Central	20 (12–30)	20 (13–26)	20 (8–36)	25 (15–53)
Edmonton	20 (9–32)	19 (11–28)	16 (7–31)	25 (14–51)
North	20 (12–32)	20 (13–27)	17 (7–33)	28 (16–56)
South	21 (14–29)	19 (12–25)	26 (16–41)	21 (14–29)
PET/CT facility				
Cross Cancer	19 (10–28)	20 (13–27)	16 (7–31)	20 (12–27)
Foothills	21 (11–29)	19 (10–25)	21 (11–35)	23 (14–41)
Royal Alex	23 (13–40)	20 (12–32)	21 (9–42)	28 (15–46)
U of A	27 (8–65)	15 (6–35)	16 (6–37)	47 (18–97)

Notes: Cross Cancer = Cross Cancer Institute; Foothills = Foothills Hospital; IQR = inter-quartile range; Royal Alex = Royal Alexandra Hospital; U of A = University of Alberta Hospital.

**Table 4:** Wait time at the first scan by priority for outpatient patients with cancer in Alberta (*n* = 8286).

Variable, Median (IQR), in Days	All Patients	P1 (Urgent)	P2 (Semi-Urgent)	P3 (Not Urgent)	P4 (Scheduled)
Patient, *n* (%)	8286	5035 (60.8)	1279 (15.4)	234 (2.8)	1738 (21)
Overall	20 (11–30)	14 (7–21)	26 (20–35)	69 (36–120)	42 (26–64)
Residence location					
Urban	20 (11–31)	14 (7–21)	26 (20–35)	74 (36–128)	41 (25–63)
Rural	21 (12–30)	15 (8–22)	26 (20–32)	40 (35–52)	46 (27–70)
Health zone					
Calgary	21 (10–29)	14 (7–22)	28 (21–37)	99 (62–141)	36 (24–58)
Central	20 (12–30)	14 (8–21)	26 (21–35)	78 (36–100)	48 (26–76)
Edmonton	20 (9–32)	13 (6–21)	25 (19–34)	37 (31–96)	54 (29–75)
North	20 (12–32)	15 (8–22)	25 (18–32)	36 (33–43)	49 (28–70)
South	21 (14–29)	18 (9–23)	28 (22–34)	44 (39–60)	36 (26–50)
PET/CT facility					
Cross Cancer	19 (10–28)	13 (6–18)	23 (17–28)	35 (29–37)	54 (30–70)
Foothills	21 (11–29)	15 (8–22)	28 (21–35)	98 (61–142)	36 (25–57)
Royal Alex	23 (13–40)	20 (12–30)	41 (27–59)	89 (69–98)	56 (16–81)
U of A	27 (8–65)	14 (6–29)	57 (35–95)	124 (79–191)	61 (12–130)

Notes: IQR = inter-quartile range; *p* = *p*-value; P1 = Priority 1, urgent; P2 = Priority 2, semi-urgent; P3 = Priority 3, non-urgent; P4 = Priority 4, planned; Alberta Health Services guidelines for wait time targets: P1: <=2 weeks; P2: <=4 weeks; P3: <=6 weeks; P4: scheduled exams, target not applicable.

**Table 5:** Summary of the associations between travel distance (in 10 km) and wait time for PET/CT in Alberta.

Analyses	Patients, *n*	Travel Distance, IRR (95% CI)	*p*
All cancers, P1–P3 priorities	6548	1.00 (1.00; 1.00)	0.108
All cancers, P1 (urgent) priority	5035	1.00 (1.00; 1.00)	0.263
All cancers, P2 (semi-urgent) priority	1279	1.00 (1.00; 1.01)	0.109
Lung cancer, P1–P3 priorities	3127	1.00 (1.00; 1.00)	0.429
Lymphoma, P1–P3 priorities	2048	1.00 (1.00; 1.00)	0.935
Prostate cancer, P1–P3 priorities	1373	1.00 (1.00; 1.01)	0.236

Notes: IRR = incident rate ratio; P1 = Priority 1, urgent; P2 = Priority 2, semi-urgent; P3 = Priority 3, not urgent; *p* = *p*-value.

## Data Availability

This study used anonymized patient-level data. The data are not publicly available because of privacy and confidentiality requirements. However, the data could be requested for research from Alberta Primary and Preventative Health Services.

## Supplementary Materials

The following supporting information can be downloaded at: https://www.mdpi.com/article/10.3390/ijerph22121816/s1, Supplementary Table S1: List of facilities with PET/CT scanner in Alberta; Supplementary Table S2: AHS PET/CT Prioritization Guidelines; Supplementary Table S3: Travel time (in minutes) to the first PET/CT scanning (*n* = 9503 patients); Supplementary Table S4: Characteristics of patients who entered the wait time cohort and patients who delayed the PET/CT scan; Supplementary Table S5: Adjusted association between wait time and travel distance for patients with lung, lymphoma, and prostate cancers with PET/CT scan (P1–P3 priorities) in Alberta (*n* = 6548); Supplementary Table S6: Adjusted association between wait time and travel distance for patients with lung, lymphoma, and prostate cancers with PET/CT scan with P1 (urgent) priority in Alberta (*n* = 5035); Supplementary Table S7: Adjusted association between wait time and travel distance for patients with lung, lymphoma, and prostate cancers with PET/CT scan with P2 (semi-urgent) priority in Alberta (*n* = 1279); Supplementary Table S8: Adjusted association between wait time and travel distance for patients with lung cancer with PET/CT scan (P1–P3 priorities) in Alberta (*n* = 3127); Supplementary Table S9: Adjusted association between wait time and travel distance for patients with lymphoma with PET/CT scan (P1–P3 priorities) in Alberta (*n* = 2048); Supplementary Table S10: Adjusted association between wait time and travel distance for patients with prostate cancer with PET/CT scan (P1–P3 priorities) in Alberta (*n* = 1373); Supplementary Table S11: Summary of the associations between travel time and wait time for PET/CT in Alberta; Supplementary Table S12: Wait time for PET/CT during pre-pandemic and COVID-19 periods in Alberta (*n* = 8286); Supplementary Table S13: Summary of the associations between travel distance (in unit of 10km) and wait time for PET/CT in Alberta for all scan priorities; Supplementary Figure S1: Alberta Health Services Zone Map.
