# Supplementary material for: Travel Distance and Its Impact on Wait Time for Positron Emission Tomography–Computed Tomography in Patients with Cancers
Source: Int J Environ Res Public Health. 2025 Dec 4;22(12):1816. doi: 10.3390/ijerph22121816 (PMC12732370; doi:10.3390/ijerph22121816)
Supplement: Supplementary file 1 [file ijerph-22-01816-s001.zip › ijerph-3848068-supplementary.pdf]

Supplementary Table S1: List of facilities with PET/CT scanner in Alberta

No	Facility name	Address
1	Cross Cancer Institute	11560 University Ave, Edmonton, AB T6G 1Z2
2	University of Alberta Hospital	8440 112 St NW, Edmonton, AB T6G 2B7
3	Royal Alexandra Hospital	10240 Kingsway NW, Edmonton, AB T5H 3V9
4	Foothills Medical Centre	1403 29 St NW, Calgary, AB T2N 2T9

Supplementary Table S2: AHS PET/CT Prioritization Guidelines

Priority	Target wait time	Purpose
Priority 1 (P1, or urgent)	< 14 days (2 weeks)	1. Staging and treatment planning of proven or strongly suspected malignancy
Priority 2 (P2, or semi-urgent)	< 28 days (4 weeks)	1. Suspected recurrence of malignancy<br>2. Characterize mass / lesion - low to moderate risk of malignancy<br>3. Myocardial Viability<br>4. Rb-82 Myocardial Perfusion Imaging
Priority 3 (P3, or not urgent)	< 42 days (6 weeks)	1. Fever of unknown origin (Outpatient)<br>2. Inflammatory investigation (e.g.: Sarcoidosis, Vasculitis)<br>3. Dementia, Focal Epilepsy
Priority 4 (P4)	Specified date	1. Scheduled exam follow up

Supplementary Table S3: Travel time (in minutes) to the first PET/CT scanning (N=9,503 patients)

Variable, median (IQR)	All patients	Lung	Lymphoma	Prostate
Overall	40 (27 – 95)	41 (27-101)	40 (27-92)	40 (27-86)
Residence location				
Urban	36 (25-56)	36 (25-63)	37 (26-55)	35 (24-52)
Rural	121 (70-170)	115 (73-170)	125 (70-172)	115 (66-167)
Health zone				
Calgary	36 (25-45)	35 (25-45)	36 (25-45)	36 (26-47)
Central	107 (79-123)	108 (81-122)	109 (79-125)	103 (74-120)
Edmonton	31 (21-39)	30 (20-39)	32 (23-40)	30 (21-38)
North	203 (115-304)	195 (118-303)	224 (124-308)	182 (99-304)
South	159 (151-208)	167 (152-208)	156 (151-208)	154 (150-208)
PET/CT facility				
Cross Cancer	44 (29-107)	48 (30-109)	41 (29-106)	45 (30-103)
Foothills	40 (28-77)	40 (28-95)	40 (27-66)	40 (27-59)
Royal Alex	35 (21-93)	36 (19-92)	37 (21-90)	35 (24-96)
U of A	34 (23-81)	30 (22-72)	33 (23-77)	39 (23-98)

Notes: Cross Cancer= Cross Cancer Institute; Foothills= Foothills Hospital; IQR= inter-quartile range; Royal Alex= Royal Alexandra Hospital; U of A= University of Alberta Hospital.

Supplementary Table S4: Characteristics of patients who entered the wait time cohort and patients who delayed the PET/CT scan

Variable	All patients	Wait time patient cohort	Delayed (excluded) patients	p
Patients, N (%)	8,791	8,286 (94.3)	505 (5.7)	
Females, n (%)	3,533 (40.2)	3,333 (40.2)	200 (39.6)	0.783
Age, in years, mean (SD)	66.8 (13.8)	66.8 (13.8)	67.2 (14.4)	0.547
Age, in years, median (IQR)	69 (61-76)	69 (61-76)	69 (61-77)	0.274
Age group, n (%)				
18-49 years	901 (10.3)	849 (10.3)	52 (10.3)	0.624
50-59 years	1,079 (12.3)	1,026 (12.4)	53 (10.5)	
60-69 years	2,529 (28.8)	2,379 (38.7)	150 (29.7)	
70-79 years	2,947 (33.5)	2,782 (33.6)	165 (32.7)	
≥80 years	1,335 (15.2)	1,250 (15.1)	85 (16.8)	
Urban residence, n (%)	7,393 (84.1)	6,963 (84.0)	430 (85.1)	0.506
Health zone, n (%)				
Calgary	3,172 (36.1)	3,073 (37.1)	99 (19.6)	<0.001
Central	1,255 (14.3)	1,180 (14.2)	75 (14.9)	
Edmonton	2,969 (33.8)	2,728 (32.9)	241 (47.7)	
North	842 (9.6)	780 (9.4)	62 (12.3)	
South	553 (6.3)	525 (6.3)	28 (5.5)	
Household income in CA$, mean (SD)	96,424 (34,979)	96,599 (35,108)	93,527 (32,673)	0.059
Comorbidities, n (%)				
Myocardial infarction	154 (1.8)	142 (1.7)	12 (2.4)	0.271
Heart failure	297 (3.4)	268 (3.2)	29 (5.7)	0.003
Peripheral vascular disease	209 (2.4)	200 (2.4)	9 (1.8)	0.366
Cerebrovascular disease	224 (2.6)	207 (2.5)	17 (3.4)	0.229
Chronic pulmonary disease	957 (10.9)	881 (10.6)	76 (15.1)	0.002
Dementia	113 (1.3)	102 (1.2)	11 (2.2)	0.067
Rheumatoid disease	144 (1.6)	141 (1.7)	3 (0.6)	0.057
Liver disease	159 (1.8)	153 (1.9)	6 (1.2)	0.281
Diabetes	1,178 (13.4)	1,105 (13.3)	73 (14.5)	0.473
Renal disease	259 (3.0)	241 (2.9)	18 (3.6)	0.398
Hemiplegia/Paraplegia	34 (0.4)	31 (0.4)	3 (0.6)	0.440
HIV/AIDS	17 (0.2)	15 (0.2)	2 (0.4)	0.286
Charlson comorbidity score, mean (SD)	3.2 (3.2)	3.2 (2.2)	3.3 (2.2)	0.352

Notes: CA$= Canadian dollars; HIV/AIDS: Human Immunodeficiency Virus/ Acquired Immunodeficiency Syndrome; IQR: interquartile range; p= p-value; SD= standard deviation.

Supplementary Table S5: Adjusted association between wait time and travel distance for patients with lung, lymphoma, and prostate cancers with PET/CT scan (P1-P3 priorities) in Alberta (N=6,548)

Variable	IRR (95% CI)	p
Travel distance (in 10km)	1.00 (1.00; 1.00)	0.108
Female	0.99 (0.95; 1.03)	0.557
Age group		
70-79 years (ref)	1.0	
18-49 years	0.88 (0.82; 0.94)	<0.001
50-59 years	0.96 (0.91; 1.01)	0.150
60-69 years	1.00 (0.96; 1.04)	0.911
≥80 years	0.99 (0.95; 1.04)	0.788
Cancer type		
Lung (ref)	1.0	
Lymphoma	0.87 (0.83; 0.91)	<0.001
Prostate	1.13 (1.07; 1.20)	<0.001
Rural residency (vs. urban)	1.00 (0.95; 1.05)	0.943
Health zone		
Calgary (ref)	1.0	
Central	1.00 (0.93; 1.08)	0.952
Edmonton	0.99 (0.91; 1.08)	0.819
North	1.01 (0.90; 1.13)	0.891
South	1.02 (0.94; 1.11)	0.607
PET/CT facility		
Cross Cancer (ref)	1.0	
Foothills	1.25 (1.15; 1.36)	<0.001
Royal Alex	1.37 (1.27; 1.48)	<0.001
U of A	1.88 (1.77; 1.99)	<0.001
Scan priority		
P1 (ref)	1.0	
P2	1.99 (1.91; 2.08)	<0.001
P3	4.89 (4.48; 5.33)	<0.001
Scan year		
2017 (ref)	1.0	
2018	1.09 (1.03; 1.15)	0.005
2019	1.06 (1.00; 1.12)	0.047
2020	0.83 (0.78; 0.88)	<0.001
2021	1.13 (1.07; 1.20)	<0.001
2022	1.21 (1.13; 1.28)	<0.001
2023	1.73 (1.55; 1.92)	<0.001
Charlson score	0.99 (0.98; 1.00)	0.008
Stage at incidence		
Stage 4 (ref)	1.0	
Not available	1.11 (1.05; 1.19)	0.001
Stage 1	1.12 (1.07; 1.17)	<0.001
Stage 2	1.10 (1.04; 1.15)	<0.001
Stage 3	1.07 (1.02; 1.13)	0.005

Notes: CI= confidence interval; Cross Cancer= Cross Cancer Institute; Foothills= Foothills Hospital; IRR= incidence rate ratio; p= p-value; P1= Priority 1, Urgent; P2= Priority 2, Semi-urgent; P3= Priority 3, non-urgent; Royal Alex= Royal Alexandra Hospital; U of A= University of Alberta Hospital.

Supplementary Table S6: Adjusted association between wait time and travel distance for patients with lung, lymphoma, and prostate cancers with PET/CT scan with P1 (urgent) priority in Alberta (N=5,035)

Variable	IRR (95% CI)	p
Travel distance (in 10km)	1.00 (1.00; 1.00)	0.263
Female	1.00 (0.96; 1.04)	0.913
Age group		
70-79 years (ref)	1.0	
18-49 years	0.85 (0.79; 0.92)	<0.001
50-59 years	0.96 (0.89; 1.02)	0.167
60-69 years	1.00 (0.95; 1.05)	0.866
≥80 years	0.97 (0.92; 1.03)	0.355
Cancer type		
Lung (ref)	1.0	
Lymphoma	0.83 (0.79; 0.88)	<0.001
Prostate	1.11 (1.04; 1.19)	0.001
Scan year		
2017 (ref)	1.0	
2018	1.15 (1.07; 1.23)	<0.001
2019	1.05 (0.98; 1.13)	0.161
2020	0.76 (0.71; 0.82)	<0.001
2021	1.13 (1.05; 1.21)	0.001
2022	1.22 (1.13; 1.31)	<0.001
2023	1.86 (1.63; 2.13)	<0.001
Charlson score	0.99 (0.98; 1.00)	0.003
Rural residency (vs. urban)	1.01 (0.96; 1.08)	0.638
Health zone		
Calgary (ref)	1.0	
Central	1.02 (0.93; 1.11)	0.727
Edmonton	1.02 (0.92; 1.13)	0.772
North	1.08 (0.95; 1.24)	0.249
South	1.05 (0.95; 1.16)	0.309
PET/CT facility		
Cross Cancer (ref)	1.0	
Foothills	1.19 (1.08; 1.31)	<0.001
Royal Alex	1.26 (1.15; 1.38)	<0.001
U of A	1.51 (1.41; 1.63)	<0.001
Stage at incidence		
Stage 4 (ref)	1.0	
Not available	1.10 (1.02; 1.19)	0.012
Stage 1	1.15 (1.09; 1.22)	<0.001
Stage 2	1.11 (1.05; 1.18)	<0.001
Stage 3	1.08 (1.02; 1.15)	0.005

Notes: CI= confidence interval; Cross Cancer= Cross Cancer Institute; Foothills= Foothills Hospital; IRR= incidence rate ratio; p= p-value; Royal Alex= Royal Alexandra Hospital; U of A= University of Alberta Hospital. The AHS target for P1 priority (urgent) is to have a scan within 2 weeks, for example, for staging and treatment planning of proven or strongly suspected malignancy.

Supplementary Table S7: Adjusted association between wait time and travel distance for patients with lung, lymphoma, and prostate cancers with PET/CT scan with P2 (semi-urgent) priority in Alberta (N=1,279)

Variable	IRR (95% CI)	p
Travel distance (in 10km)	1.00 (1.00; 1.01)	0.109
Female	1.00 (1.00; 1.01)	0.980
Age group		
70-79 years (ref)	1.0	
18-49 years	1.04 (0.92; 1.18)	0.533
50-59 years	1.02 (0.93; 1.12)	0.688
60-69 years	1.00 (0.94; 1.07)	0.892
≥80 years	1.04 (0.95; 1.13)	0.422
Cancer type		
Lung (ref)	1.0	
Lymphoma	0.95 (0.89; 1.02)	0.199
Prostate	1.04 (0.96; 1.13)	0.363
Scan year		
2017 (ref)	1.0	
2018	0.95 (0.86; 1.05)	0.292
2019	1.08 (0.98; 1.19)	0.136
2020	1.02 (0.92; 1.12)	0.753
2021	0.98 (0.88; 1.09)	0.753
2022	1.13 (1.02; 1.25)	0.024
2023	1.52 (1.28; 1.80)	<0.001
Rural residency (vs. urban)	0.97 (0.90; 1.06)	0.533
Health zone		
Calgary (ref)	1.0	
Central	0.98 (0.84; 1.14)	0.792
Edmonton	0.92 (0.79; 1.08)	0.318
North	0.84 (0.70; 1.01)	0.062
South	0.90 (0.76; 1.06)	0.219
PET/CT facility		
Cross Cancer (ref)	1.0	
Foothills	1.22 (1.05; 1.42)	0.010
Royal Alex	1.70 (1.49; 1.93)	<0.001
U of A	2.83 (2.60; 3.08)	<0.001

Notes: CI= confidence interval; Cross Cancer= Cross Cancer Institute; Foothills= Foothills Hospital; IRR= incidence rate ratio; p= p-value; U of A= University of Alberta Hospital. AHS target for P2 priority is to have scan within 4 weeks. For example, suspected recurrence of malignancy, characterize mass / lesion - low to moderate risk of malignancy, myocardial viability, Rb-82 Myocardial Perfusion Imaging.

Supplementary Table S8: Adjusted association between wait time and travel distance for patients with lung cancer with PET/CT scan (P1-P3 priorities) in Alberta (N=3,127)

Variable	IRR (95% CI)	p
Travel distance (in 10km)	1.00 (1.00; 1.00)	0.429
Female	1.01 (0.96; 1.05)	0.807
Age group		
70-79 years (ref)	1.0	
18-49 years	0.88 (0.76; 1.03)	0.108
50-59 years	0.92 (0.85; 0.99)	0.032
60-69 years	0.97 (0.92; 1.02)	0.194
≥80 years	1.01 (0.95; 1.08)	0.681
Rural residency (vs. urban)	0.98 (0.92; 1.04)	0.510
Health zone		
Calgary (ref)	1.0	
Central	1.03 (0.93; 1.14)	0.542
Edmonton	1.00 (0.89; 1.12)	0.976
North	1.08 (0.93; 1.26)	0.332
South	1.03 (0.93; 1.16)	0.545
PET/CT facility		
Cross Cancer (ref)	1.0	
Foothills	1.12 (1.01; 1.25)	0.034
Royal Alex	1.25 (1.13; 1.38)	<0.001
U of A	1.55 (1.43; 1.69)	<0.001
Scan priority		
P1 (ref)	1.0	
P2	1.79 (1.69; 1.89)	<0.001
P3	2.45 (2.10; 2.86)	<0.001
Scan year		
2017 (ref)	1.0	
2018	1.09 (1.01; 1.18)	0.026
2019	1.07 (0.98; 1.15)	0.117
2020	0.85 (0.78; 0.92)	<0.001
2021	1.18 (1.09; 1.28)	<0.001
2022	1.18 (1.08; 1.28)	<0.001
2023	1.75 (1.50; 2.06)	<0.001
Charlson score	0.99 (0.98; 1.00)	0.054
Stage at incidence		
Stage 4 (ref)	1.0	
Not available	1.08 (0.98; 1.20)	0.104
Stage 1	1.16 (1.10; 1.23)	<0.001
Stage 2	1.13 (1.04; 1.22)	0.004
Stage 3	1.08 (1.01; 1.15)	0.017

Notes: CI= confidence interval; Cross Cancer= Cross Cancer Institute; Foothills= Foothills Hospital; IRR= incidence rate ratio; p= p-value; P1= Priority 1, Urgent; P2= Priority 2, Semi-urgent; P3= Priority 3, non-urgent; Royal Alex= Royal Alexandra Hospital; U of A= University of Alberta Hospital.

Supplementary Table S9: Adjusted association between wait time and travel distance for patients with lymphoma with PET/CT scan (P1-P3 priorities) in Alberta (N=2,048)

Variable	IRR (95% CI)	p
Travel distance (in 10km)	1.00 (1.00; 1.00)	0.935
Female	0.97 (0.92; 1.04)	0.419
Age group		
70-79 years (ref)	1.0	
18-49 years	0.89 (0.82; 0.98)	0.014
50-59 years	0.93 (0.84; 1.03)	0.148
60-69 years	1.00 (0.91; 1.09)	0.954
≥80 years	0.97 (0.86; 1.10)	0.662
Rural residence (vs. urban)	1.08 (0.97; 1.19)	0.152
Health zone		
Calgary (ref)	1.0	
Central	1.08 (0.93; 1.26)	0.295
Edmonton	1.05 (0.89; 1.25)	0.560
North	1.02 (0.81; 1.28)	0.863
South	1.20 (1.02; 1.42)	0.027
PET/CT facility		
Cross Cancer (ref)	1.0	
Foothills	1.44 (1.23; 1.68)	<0.001
Royal Alex	1.58 (1.27; 1.96)	<0.001
U of A	1.54 (1.35; 1.75)	<0.001
Scan priority		
P1 (ref)	1.0	
P2	2.22 (2.04; 2.40)	<0.001
P3	3.94 (3.07; 5.05)	<0.001
Scan year		
2017 (ref)	1.0	
2018	1.17 (1.05; 1.30)	0.004
2019	1.07 (0.97; 1.19)	0.194
2020	0.73 (0.66; 0.81)	<0.001
2021	0.99 (0.89; 1.10)	0.794
2022	1.03 (0.90; 1.17)	0.670
2023	1.54 (1.19; 2.00)	0.001
Stage at incidence		
Stage 4 (ref)	1.0	
Not available	1.15 (1.01; 1.31)	0.036
Stage 1	1.07 (0.98; 1.17)	0.126
Stage 2	1.05 (0.96; 1.14)	0.268
Stage 3	1.05 (0.96; 1.14)	0.325
Time from diagnosis to index (month)	1.00 (1.00; 1.00)	0.018

Notes: CI= confidence interval; Cross Cancer= Cross Cancer Institute; Foothills= Foothills Hospital; IRR= incidence rate ratio; p= p-value; P1= Priority 1, Urgent; P2= Priority 2, Semi-urgent; P3= Priority 3, non-urgent; Royal Alex= Royal Alexandra Hospital; U of A= University of Alberta Hospital.

Supplementary Table S10: Adjusted association between wait time and travel distance for patients with prostate cancer with PET/CT scan (P1-P3 priorities) in Alberta (N=1,373)

Variable	IRR (95% CI)	p
Travel distance (in 10km)	1.00 (1.00; 1.01)	0.236
Age group		
70-79 years (ref)	1.0	
18-49 years	0.92 (0.61; 1.40)	0.696
50-59 years	1.09 (0.94; 1.25)	0.244
60-69 years	1.08 (0.99; 1.18)	0.075
≥80 years	1.00 (0.92; 1.10)	0.931
Rural residence (vs. urban)	0.94 (0.85; 1.05)	0.265
Health zone		
Calgary (ref)	1.0	
Central	0.93 (0.79; 1.11)	0.419
Edmonton	0.99 (0.81; 1.20)	0.879
North	0.95 (0.76; 1.18)	0.624
South	0.84 (0.69; 1.02)	0.080
PET/CT facility		
Cross Cancer (ref)	1.0	
Foothills	1.28 (1.06; 1.55)	0.011
Royal Alex	1.34 (1.16; 1.56)	<0.001
U of A	2.19 (1.97; 2.43)	<0.001
Scan priority		
P1 (ref)	1.0	
P2	1.95 (1.79; 2.13)	<0.001
P3	6.13 (5.45; 6.89)	<0.001
Scan year		
2017 (ref)	1.0	
2018	1.05 (0.91; 1.21)	0.490
2019	1.15 (1.01; 1.32)	0.039
2020	1.08 (0.94; 1.25)	0.274
2021	1.37 (1.20; 1.56)	<0.001
2022	1.56 (1.37; 1.79)	<0.001
2023	2.06 (1.71; 2.49)	<0.001

Notes: CI= confidence interval; Cross Cancer= Cross Cancer Institute; Foothills= Foothills Hospital; IRR= incidence rate ratio; p= p-value; P1= Priority 1, Urgent; P2= Priority 2, Semi-urgent; P3= Priority 3, Royal Alex= Royal Alexandra Hospital; U of A= University of Alberta Hospital.

Supplementary Table S11: Summary of the associations between travel time and wait time for PET/CT in Alberta

Analyses	Patients, n	Travel time (in unit of 10 minutes), IRR (95% CI)	p
All cancers, P1-P3 priorities	6,548	1.00 (1.00; 1.01)	0.159
All cancers, P1 (urgent) priority	5,035	1.00 (1.00; 1.01)	0.367
All cancers, P2 (semi-urgent) priority	1,279	1.00 (1.00; 1.01)	0.122
Lung cancer, P1-P3 priorities	3,127	1.00 (1.00; 1.01)	0.515
Lymphoma, P1-P3 priorities	2,048	1.00 (0.99; 1.01)	0.917
Prostate cancer, P1-P3 priorities	1,373	1.00 (1.00; 1.01)	0.292

Notes: IRR= incident rate ratio; P1= Priority 1, Urgent; P2= Priority 2, Semi-urgent; P3= Priority 3, non-urgent; P4= Priority 4, planned; p= p-value.

Supplementary Table S12: Wait time for PET/CT during pre-pandemic and COVID-19 periods in Alberta (N=8,286)

Variable, median (IQR)	All patients	Pre-pandemic period (N=4,447)	COVID-19 period (N=3,839)	p
Overall	20 (11-30)	20 (12-30)	21 (9-31)	0.209
Residence location				
Urban	20 (11-31)	20 (12-30)	21 (9-31)	0.403
Rural	21 (12-30)	21 (13-30)	20 (10-30)	0.200
Health zone				
Calgary	21 (10-29)	20 (11-30)	21 (9-29)	0.402
Central	20 (12-30)	21 (13-32)	19 (9-30)	0.013
Edmonton	20 (9-32)	20 (12-31)	20 (7-33)	0.112
North	20 (12-32)	20 (13-30)	20 (10-34)	0.888
South	21 (14-29)	21 (14-28)	22 (13-29)	0.829
PET/CT facility				
Cross Cancer	19 (10-28)	20 (12-28)	17 (7-27)	<0.001
Foothills	21 (11-29)	20 (12-30)	21 (10-28)	0.682
Royal Alex	23 (13-40)	--	23 (13-40)	--
U of A	27 (8-65)	25 (8-70)	27 (9-62)	0.490

Notes: Cross Cancer= Cross Cancer Institute; Foothills= Foothills Hospital; IQR= inter-quartile range; p= p-value.

Supplementary Table S13: Summary of the associations between travel distance (in unit of 10km) and wait time for PET/CT in Alberta for all scan priorities

Analyses	Patients, n	Travel distance, IRR (95% CI)	p
All cancers, all priority levels (P1-P4)	8,286	1.00 (1.00; 1.00)	0.107
All cancers, P1 priority	5,035	1.00 (1.00; 1.00)	0.263
All cancers, P2 priority	1,279	1.00 (1.00; 1.01)	0.109
All cancers, P3 priority	234	0.99 (0.98; 1.00)	0.019
All cancers, P4 priority	1,738	1.00 (0.99; 1.00)	0.820
Lung cancer, all priority levels (P1-P4)	3,613	1.00 (1.00; 1.00)	0.950
Lymphoma, all priority levels (P1-P4)	3,007	1.00 (1.00; 1.00)	0.823
Prostate cancer, all priority levels (P1-P4)	1,666	1.01 (1.00; 1.01)	0.034

Notes: IRR= incident rate ratio; P1= Priority 1, Urgent; P2= Priority 2, Semi-urgent; P3= Priority 3, non-urgent; P4= Priority 4, planned; p= p-value.

Supplementary Figure S1: Alberta Health Services Zone Map

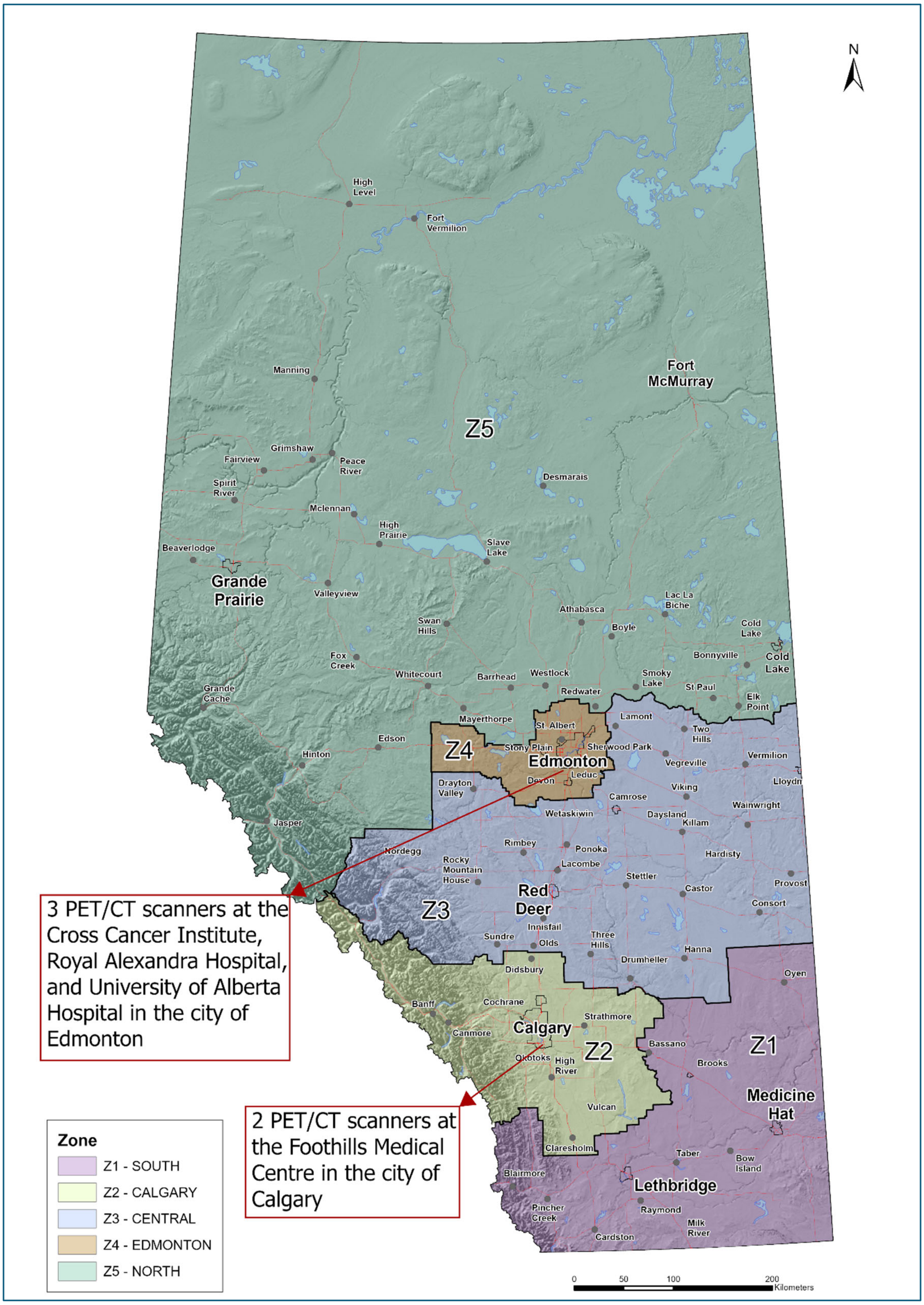

Alberta population count in 2021

Health zone	Population count	% total population	Land mass (km^2)
North	485,425	10.9	448,500
Edmonton	1,435,318	32.3	11,800
Central	481,135	10.8	95,500
Calgary	1,726,570	38.9	39,300
South	314,430	7.1	65,500
**Total**	**4,442,879**	**100**	
